# The genome sequence of the surf clam, *Spisula solida* (Linnaeus, 1758)

**DOI:** 10.12688/wellcomeopenres.19486.1

**Published:** 2023-05-24

**Authors:** Anna Holmes, Marine Biological Association Genome Acquisition Lab, Darwin Tree of Life Barcoding collective, Wellcome Sanger Institute Tree of Life programme, Wellcome Sanger Institute Scientific Operations: DNA Pipelines collective, Tree of Life Core Informatics collective, Darwin Tree of Life Consortium

**Affiliations:** 1 Amgueddfa Cymru, Cardiff, Wales, UK

**Keywords:** Spisula solida, surf clam, genome sequence, chromosomal, Venerida

## Abstract

We present a genome assembly from an individual *Spisula solida* (the surf clam; Mollusca; Bivalvia; Venerida; Mactridae). The genome sequence is 932.1 megabases in span. Most of the assembly is scaffolded into 19 chromosomal pseudomolecules. The mitochondrial genome has also been assembled and is 19.3 kilobases in length. Gene annotation of this assembly on Ensembl identified 13,833 protein coding genes.

## Species taxonomy

Eukaryota; Metazoa; Spiralia; Lophotrochozoa; Mollusca; Bivalvia; Autobranchia; Heteroconchia; Euheterodonta; Imparidentia; Neoheterodontei; Venerida; Mactroidea; Mactridae; *Spisula*; *Spisula solida* (Linnaeus, 1758) (NCBI:txid31201).

## Background

Surf clams (Mactridae) are commonly eaten worldwide and are an important fishery resource. *Spisula solida* is one of three British *Spisula* species found on clean, sandy, often exposed beaches, living buried low in the intertidal zone and offshore. It is a filter feeder, preferring clean, sandy environments and avoiding muddy sediments. Its range extends from Norway and south to Morocco, but excluding the Mediterranean. Instead, *Spisula elliptica* and *Spisula subtruncata* are recorded from the Mediterranean ( GBIF). Another species, *Spisula solidissima*, has been documented in Britain; however it is an imported species originating from the USA.

*Spisula solida*, solid-shelled and triangular in outline, can grow up to 45 mm in length. It has a creamy shell covered with a thin brown periostracum that flakes off easily. The growth lines are clear, with numerous fine concentric lines between each pair of growth lines ( Amguddfa Cymru - National Museum Wales, 2016). *Spisula* species have thicker shells than other genera within the family such as *Mactra* and the internal dentition can aid in their separation. The lateral teeth are serrated in *Spisula* but not in *Mactra*, and the length and angle of the cardinal teeth serve as a diagnostic character.

The genome of the surf clam, *Spisula solida*, was sequenced as part of the Darwin Tree of Life Project; a collaborative effort to sequence all named eukaryotic species in the Atlantic Archipelago of Britain and Ireland.

## Genome sequence report

**Figure 1.**
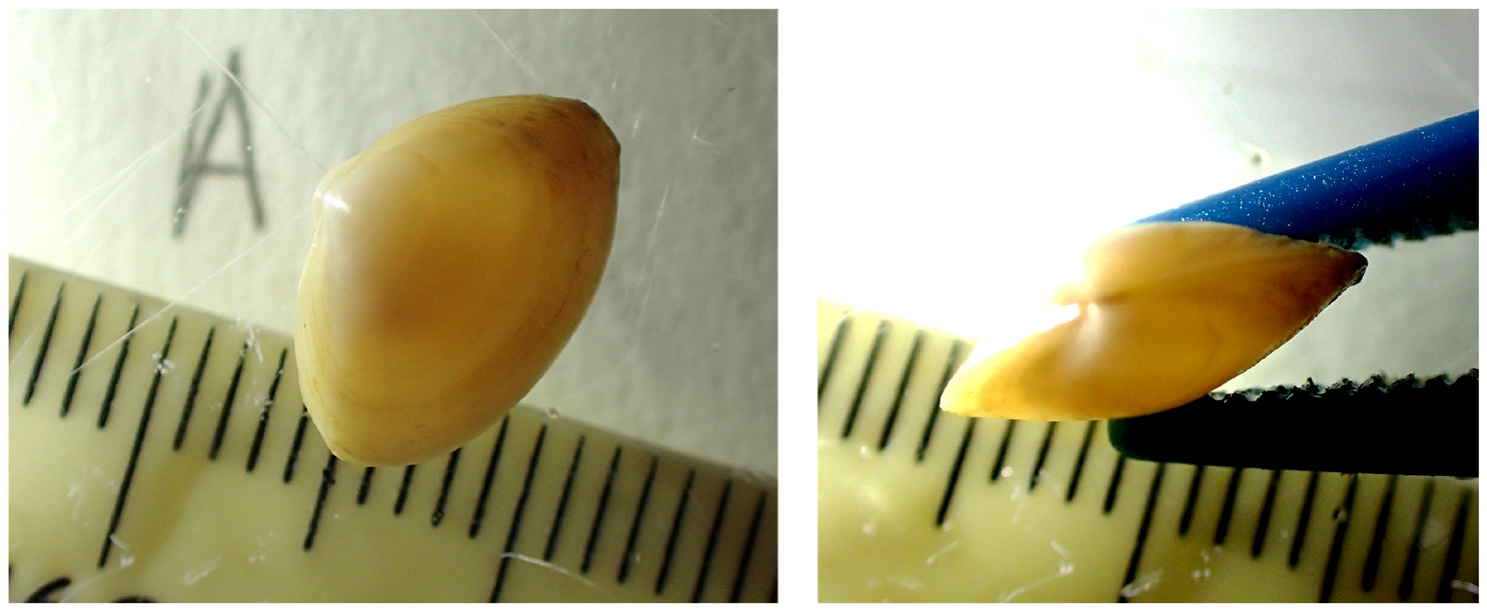
Photographs of the *Spisula solida* (xbSpiSoli1) specimen used for genome sequencing.

The genome was sequenced from one *Spisula solida* specimen ( Figure 1) collected from Jennycliff Bay, Plymouth, UK. A total of 31-fold coverage in Pacific Biosciences single-molecule HiFi long reads was generated. Primary assembly contigs were scaffolded with chromosome conformation Hi-C data. Manual assembly curation corrected 35 missing or mis-joins and removed four haplotypic duplications, reducing the scaffold number by 32.14%, and increasing the scaffold N50 by 1.37%.

**Figure 2.**
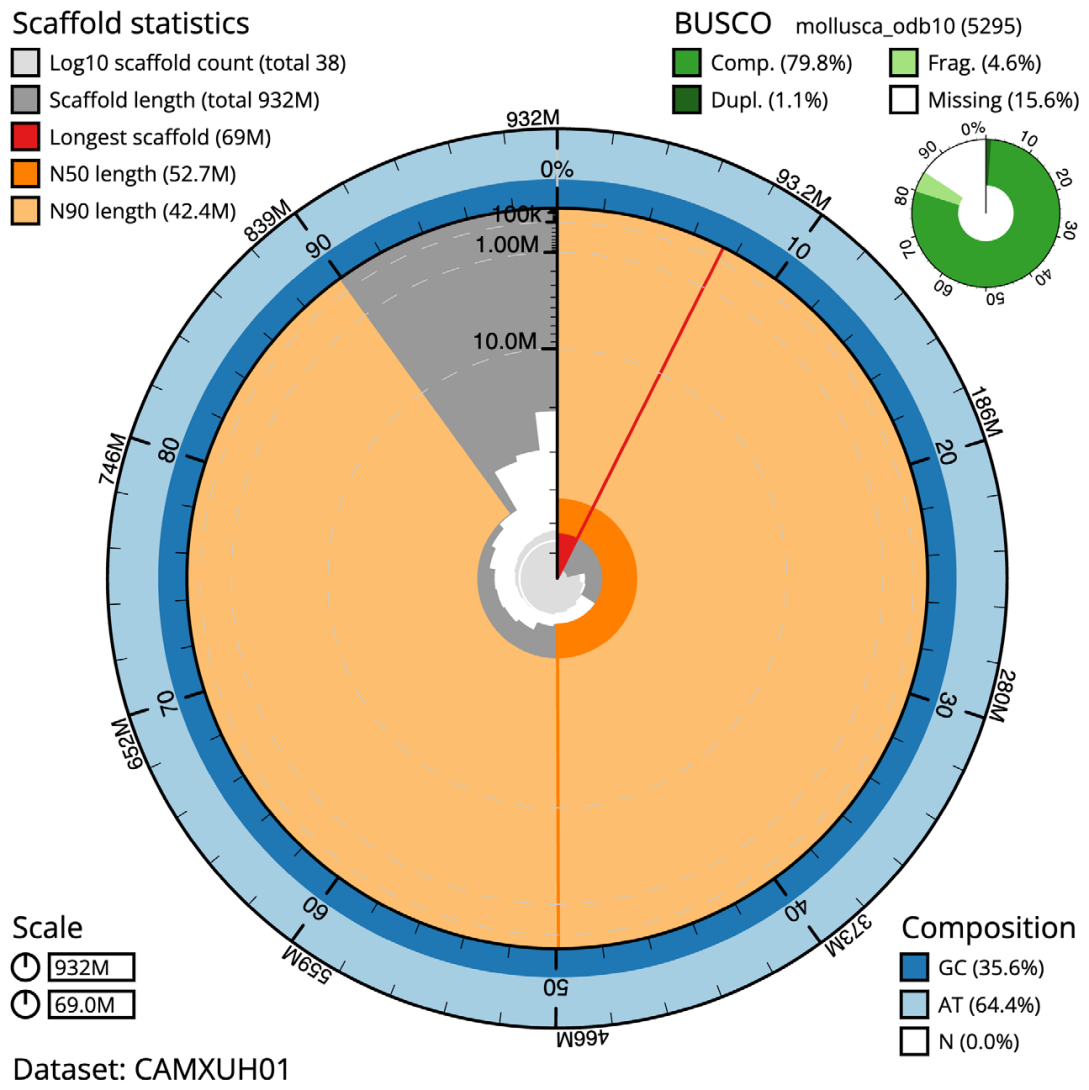
Genome assembly of *Spisula solida*, xbSpiSoli1.1: metrics. The BlobToolKit Snailplot shows N50 metrics and BUSCO gene completeness. The main plot is divided into 1,000 size-ordered bins around the circumference with each bin representing 0.1% of the 932,108,243 bp assembly. The distribution of scaffold lengths is shown in dark grey with the plot radius scaled to the longest scaffold present in the assembly (69,035,870 bp, shown in red). Orange and pale-orange arcs show the N50 and N90 scaffold lengths (52,695,421 and 42,436,359 bp), respectively. The pale grey spiral shows the cumulative scaffold count on a log scale with white scale lines showing successive orders of magnitude. The blue and pale-blue area around the outside of the plot shows the distribution of GC, AT and N percentages in the same bins as the inner plot. A summary of complete, fragmented, duplicated and missing BUSCO genes in the mollusca_odb10 set is shown in the top right. An interactive version of this figure is available at https://blobtoolkit.genomehubs.org/view/xbSpiSoli1.1/dataset/CAMXUH01/snail.

The final assembly has a total length of 932.1 Mb in 38 sequence scaffolds with a scaffold N50 of 52.7 Mb ( Table 1). Most (99.95%) of the assembly sequence was assigned to 19 chromosomal-level scaffolds. Chromosome-scale scaffolds confirmed by the Hi-C data are named in order of size ( Figure 2– Figure 5; Table 2). While not fully phased, the assembly deposited is of one haplotype. Contigs corresponding to the second haplotype have also been deposited. The mitochondrial genome was also assembled and can be found as a contig within the multifasta file of the genome submission.

**Table 1.** Genome data for *Spisula solida*, xbSpiSoli1.1.

Project accession data		
Assembly identifier	xbSpiSoli1.1	
Species	*Spisula solida*	
Specimen	xbSpiSoli1	
NCBI taxonomy ID	31201	
BioProject	PRJEB56104	
BioSample ID	SAMEA8724796	
Isolate information	xbSpiSoli1 (DNA sequencing) xbSpiSoli2 (Hi-C scaffolding)	
Assembly metrics *		*Benchmark*
Consensus quality (QV)	62.8	*≥ 50*
*k*-mer completeness	100%	*≥ 95%*
BUSCO **	C:79.8%[S:78.7%,D:1.1%], F:4.6%,M:15.6%,n:5,295	*C ≥ 95%*
Percentage of assembly mapped to chromosomes	99.95%	*≥ 95%*
Sex chromosomes	-	*localised homologous pairs*
Organelles	Mitochondrial genome assembled	*complete single alleles*
Raw data accessions		
PacificBiosciences SEQUEL II	ERR10287555, ERR10287556	
Hi-C Illumina	ERR10297838	
Genome assembly		
Assembly accession	GCA_947247005.1	
*Accession of alternate haplotype*	GCA_947247015.1	
Span (Mb)	932.1	
Number of contigs	132	
Contig N50 length (Mb)	15	
Number of scaffolds	38	
Scaffold N50 length (Mb)	52.7	
Longest scaffold (Mb)	69.0	
Genome annotation		
Number of protein-coding genes	13,833	
Number of non-coding genes	8,506	
Number of gene transcripts	31,615	

* Assembly metric benchmarks are adapted from column VGP-2020 of “Table 1: Proposed standards and metrics for defining genome assembly quality” from ( Rhie *et al.*, 2021).

** BUSCO scores based on the mollusca_odb10 BUSCO set using v5.3.2. C = complete [S = single copy, D = duplicated], F = fragmented, M = missing, n = number of orthologues in comparison. A full set of BUSCO scores is available at https://blobtoolkit.genomehubs.org/view/xbSpiSoli1.1/dataset/CAMXUH01/busco.

**Figure 3.**
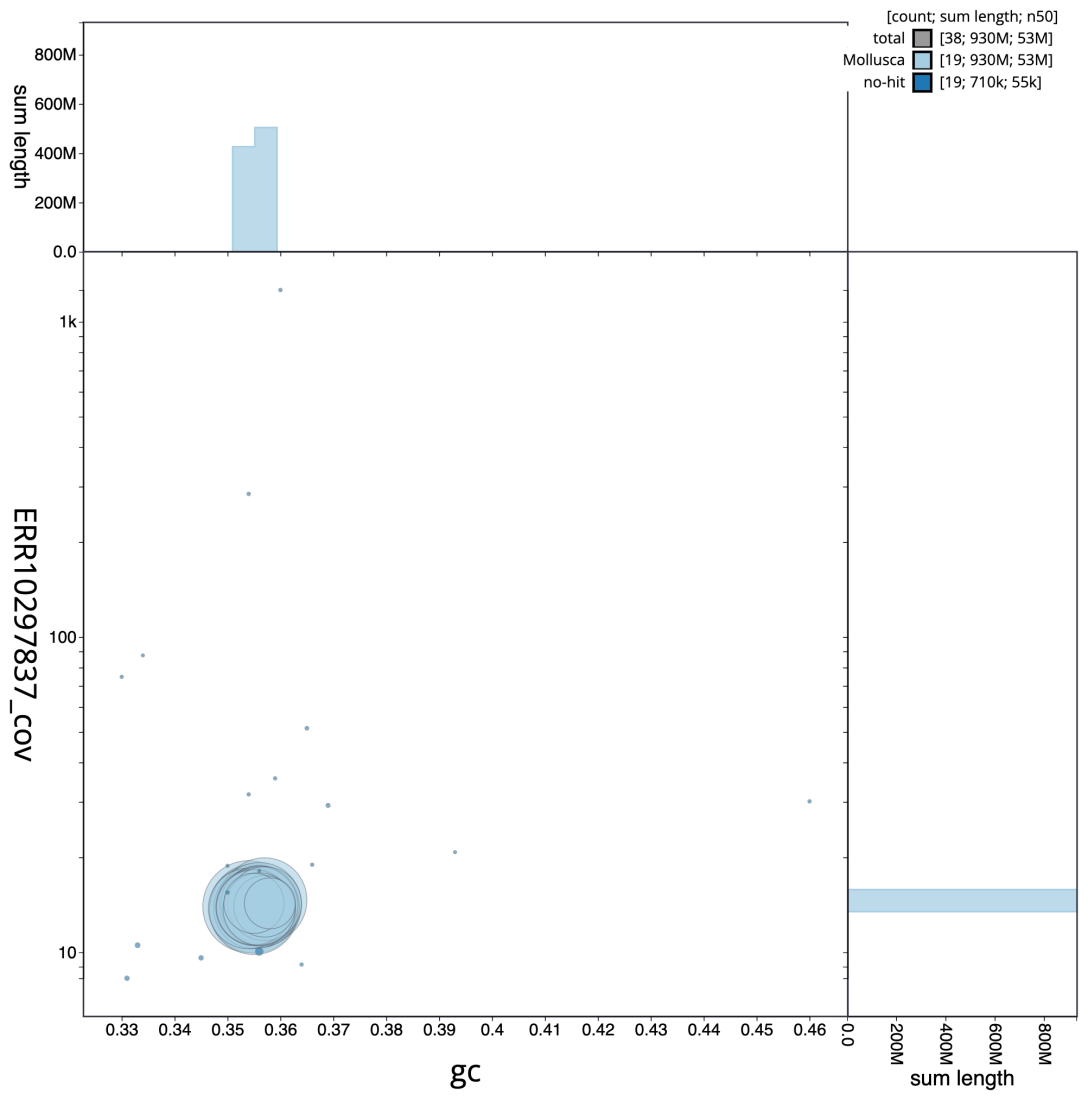
Genome assembly of *Spisula solida*, xbSpiSoli1.1: GC coverage. BlobToolKit GC-coverage plot. Scaffolds are coloured by phylum. Circles are sized in proportion to scaffold length. Histograms show the distribution of scaffold length sum along each axis. An interactive version of this figure is available at https://blobtoolkit.genomehubs.org/view/xbSpiSoli1.1/dataset/CAMXUH01/blob.

**Figure 4.**
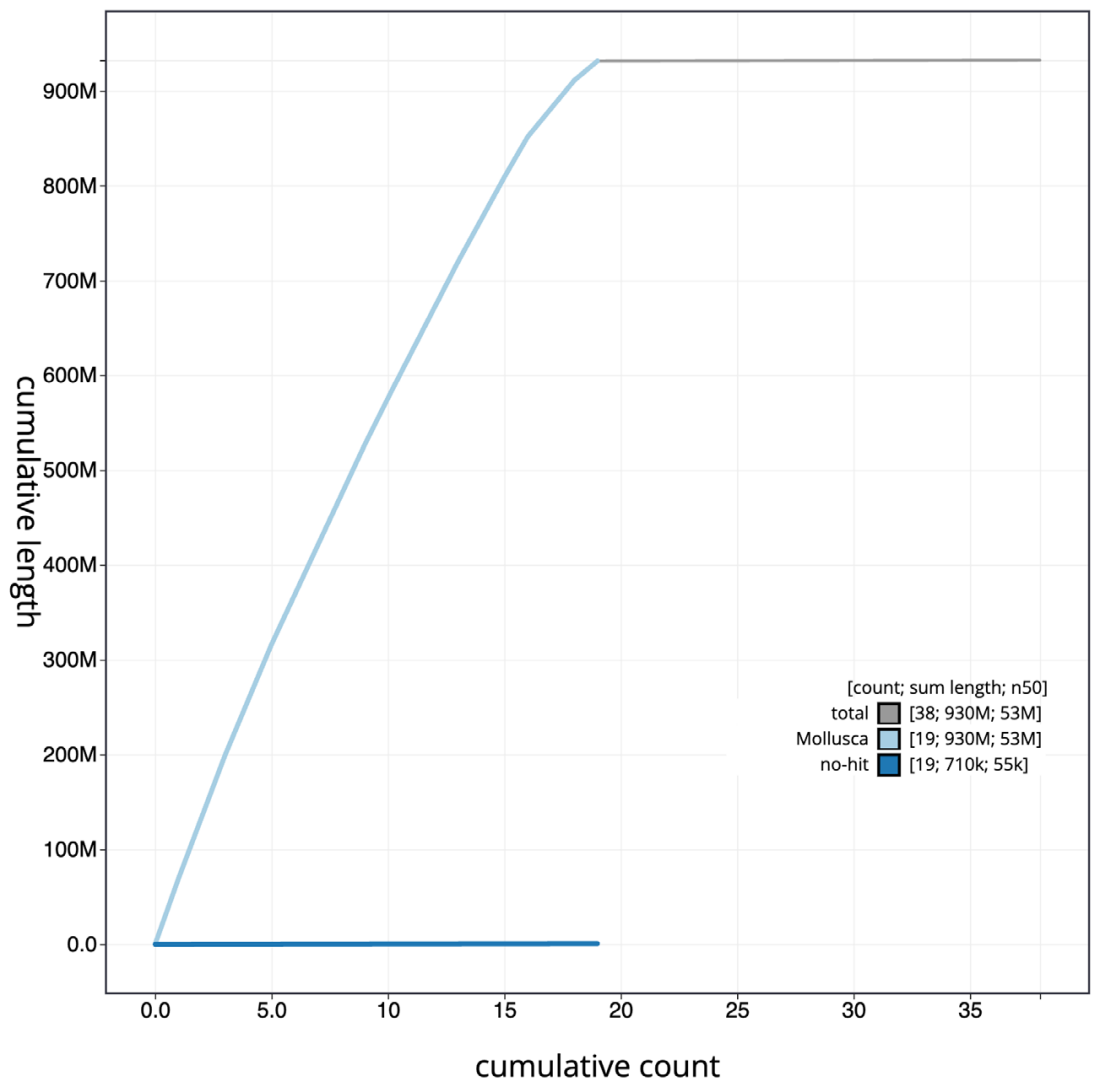
Genome assembly of *Spisula solida*, xbSpiSoli1.1: cumulative sequence. BlobToolKit cumulative sequence plot. The grey line shows cumulative length for all scaffolds. Coloured lines show cumulative lengths of scaffolds assigned to each phylum using the buscogenes taxrule. An interactive version of this figure is available at https://blobtoolkit.genomehubs.org/view/xbSpiSoli1.1/dataset/CAMXUH01/cumulative.

**Figure 5.**
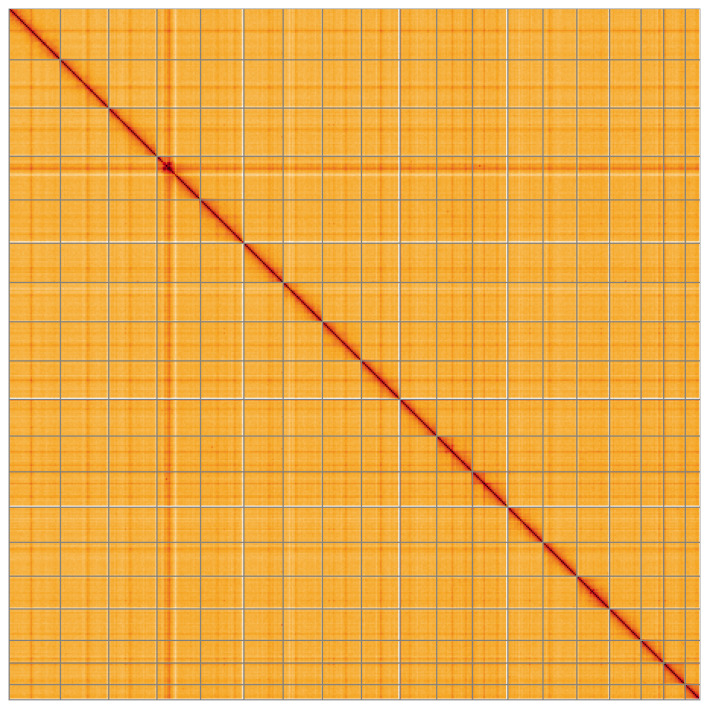
Genome assembly of *Spisula solida*, xbSpiSoli1.1: Hi-C contact map. Hi-C contact map of the xbSpiSoli1.1 assembly, visualised using HiGlass. Chromosomes are shown in order of size from left to right and top to bottom. An interactive version of this figure is available at https://genome-note-higlass.tol.sanger.ac.uk/l/?d=bf1x8DCYTLO3Bnq58LJnXA.

**Table 2.** Chromosomal pseudomolecules in the genome assembly of *Spisula solida*, xbSpiSoli1.

INSDC accession	Chromosome	Size (Mb)	GC%
OX365944.1	1	69.04	35.4
OX365945.1	2	65.07	35.5
OX365946.1	3	65	35.5
OX365947.1	4	58.72	35.7
OX365948.1	5	58.36	35.6
OX365949.1	6	52.7	35.6
OX365950.1	7	52.92	35.5
OX365951.1	8	52.89	35.4
OX365952.1	9	51.99	35.6
OX365953.1	10	49.31	35.6
OX365954.1	11	48.23	35.6
OX365955.1	12	47.69	35.6
OX365956.1	13	47.32	35.5
OX365957.1	14	45.58	35.5
OX365958.1	15	44.18	35.7
OX365959.1	16	42.44	35.6
OX365960.1	17	30.44	35.7
OX365961.1	18	29.02	35.5
OX365962.1	19	20.52	35.8
OX365963.1	MT	0.02	35.3
-	unplaced	0.69	35.4

The estimated Quality Value (QV) of the final assembly is 62.8 with *k*-mer completeness of 100%, and the assembly has a BUSCO v5.3.2completeness of 79.8% (single = 78.7%, duplicated = 1.1%), using the mollusca_odb10 reference set ( *n* = 5,295).

Metadata for specimens, spectral estimates, sequencing runs, contaminants and pre-curation assembly statistics can be found at https://links.tol.sanger.ac.uk/species/31201.

## Genome annotation report

The *Spisula solida* genome assembly GCA_947247005.1 was annotated using the Ensembl rapid annotation pipeline ( Table 1; https://rapid.ensembl.org/Spisula_solida_GCA_947247005.1/Info/Index). The resulting annotation includes 31,615 transcribed mRNAs from 13,833 protein-coding and 8,506 non-coding genes.

## Methods

### Sample acquisition and nucleic acid extraction

Two *Spisula solida* individuals (specimen numbers MBA-201117–015A and MBA-201117–015B, ToLIDs xbSpiSoli1 and xbSpiSoli2) were collected from Jennycliff Bay, Plymouth, UK (latitude 50.34, longitude –4.13) on 17 November 2020. The specimens were retrieved from sand using a grab sampler (MV Sepia). The specimens were collected and identified by Anna Holmes (Amgueddfa Cymru) and then flash-frozen in liquid nitrogen prior to sample processing.

DNA was extracted at the Tree of Life laboratory, Wellcome Sanger Institute (WSI). The xbSpiSoli1 sample was weighed and dissected on dry ice. The tissue was disrupted using a Nippi Powermasher fitted with a BioMasher pestle. High molecular weight (HMW) DNA was extracted using the Qiagen MagAttract HMW DNA extraction kit. HMW DNA was sheared into an average fragment size of 12–20 kb in a Megaruptor 3 system with speed setting 30. Sheared DNA was purified by solid-phase reversible immobilisation using AMPure PB beads with a 1.8X ratio of beads to sample to remove the shorter fragments and concentrate the DNA sample. The concentration of the sheared and purified DNA was assessed using a Nanodrop spectrophotometer and Qubit Fluorometer and Qubit dsDNA High Sensitivity Assay kit. Fragment size distribution was evaluated by running the sample on the FemtoPulse system.

### Sequencing

Pacific Biosciences HiFi circular consensus DNA sequencing libraries were constructed according to the manufacturers’ instructions. DNA sequencing was performed by the Scientific Operations core at the WSI on Pacific Biosciences SEQUEL II (HiFi) instrument. Hi-C data were also generated from tissue of xbSpiSoli2 using the Arima2 kit and sequenced on the Illumina NovaSeq 6000 instrument.

### Genome assembly, curation and evaluation

Assembly was carried out with Hifiasm ( Cheng *et al.*, 2021) and haplotypic duplication was identified and removed with purge_dups ( Guan *et al.*, 2020). The assembly was then scaffolded with Hi-C data ( Rao *et al.*, 2014) using YaHS ( Zhou *et al.*, 2023). The assembly was checked for contamination and corrected using the gEVAL system ( Chow *et al.*, 2016) as described previously ( Howe *et al.*, 2021). Manual curation was performed using gEVAL,HiGlass ( Kerpedjiev *et al.*, 2018) and Pretext ( Harry, 2022). The mitochondrial genome was assembled using MitoHiFi ( Uliano-Silva *et al.*, 2022), which performed annotation using MitoFinder ( Allio *et al.*, 2020).

A Hi-C map for the final assembly was produced using bwa-mem2 ( Vasimuddin *et al.*, 2019) in the Cooler file format ( Abdennur & Mirny, 2020). To assess the assembly metrics, the *k*-mer completeness and QV consensus quality values were calculated in Merqury ( Rhie *et al.*, 2020). This work was done using Nextflow ( Di Tommaso *et al.*, 2017) DSL2 pipelines “sanger-tol/readmapping” ( Surana *et al.*, 2023a) and “sanger-tol/genomenote” ( Surana *et al.*, 2023b). The genome was analysed within the BlobToolKit environment ( Challis *et al.*, 2020) and BUSCO scores ( Manni *et al.*, 2021; Simão *et al.*, 2015) were calculated.

Table 3 contains a list of relevant software tool versions and sources.

**Table 3.** Software tools: versions and sources.

Software tool	Version	Source
BlobToolKit	4.0.7	https://github.com/blobtoolkit/blobtoolkit
BUSCO	5.3.2	https://gitlab.com/ezlab/busco
gEVAL	N/A	https://geval.org.uk/
Hifiasm	0.16.1-r375	https://github.com/chhylp123/hifiasm
HiGlass	1.11.6	https://github.com/higlass/higlass
Merqury	MerquryFK	https://github.com/thegenemyers/MERQURY.FK
MitoHiFi	2	https://github.com/marcelauliano/MitoHiFi
PretextView	0.2	https://github.com/wtsi-hpag/PretextView
purge_dups	1.2.3	https://github.com/dfguan/purge_dups
YaHS	yahs-1.1.91eebc2	https://github.com/c-zhou/yahs

### Genome annotation

The Ensembl gene annotation system ( Aken *et al.*, 2016) was used to generate annotation for the *Spisula solida* assembly (GCA_947247005.1). Annotation was created primarily through alignment of transcriptomic data to the genome, with gap filling via protein-to-genome alignments of a select set of proteins from UniProt ( UniProt Consortium, 2019).

### Legal and ethical review process for Darwin Tree of Life Partner submitted materials

The materials that have contributed to this genome note have been supplied by a Darwin Tree of Life Partner.

The submission of materials by a Darwin Tree of Life Partner is subject to the **‘Darwin Tree of Life Project Sampling Code of Practice’**,which can be found in full on the Darwin Tree of Life website here. By agreeing with and signing up to the Sampling Code of Practice, the Darwin Tree of Life Partner agrees they will meet the legal and ethical requirements and standards set out within this document in respect of all samples acquired for, and supplied to, the Darwin Tree of Life Project.

Further, the Wellcome Sanger Institute employs a process whereby due diligence is carried out proportionate to the nature of the materials themselves, and the circumstances under which they have been/are to be collected and provided for use. The purpose of this is to address and mitigate any potential legal and/or ethical implications of receipt and use of the materials as part of the research project, and to ensure that in doing so we align with best practice wherever possible.

The overarching areas of consideration are:

- Ethical review of provenance and sourcing of the material
- Legality of collection, transfer and use (national and international)

Each transfer of samples is further undertaken according to a Research Collaboration Agreement or Material Transfer Agreement entered into by the Darwin Tree of Life Partner, Genome Research Limited (operating as the Wellcome Sanger Institute), and in some circumstances other Darwin Tree of Life collaborators.

## Data Availability

European Nucleotide Archive: *Spisula solida* (a clam). Accession number PRJEB56104; https://identifiers.org/ena.embl/PRJEB56104. ( Wellcome Sanger Institute, 2022) The genome sequence is released openly for reuse. The *Spisula solida* genome sequencing initiative is part of the Darwin Tree of Life (DToL) project. All raw sequence data and the assembly have been deposited in INSDC databases. Raw data and assembly accession identifiers are reported in Table 1.

## Author information

Members of the Marine Biological Association Genome Acquisition Lab are listed here:

https://doi.org/10.5281/zenodo.4783604.

Members of the Darwin Tree of Life Barcoding collective are listed here: https://doi.org/10.5281/zenodo.4893703.

Members of the Wellcome Sanger Institute Tree of Life programme are listed here: https://doi.org/10.5281/zenodo.4783585.

Members of Wellcome Sanger Institute Scientific Operations: DNA Pipelines collective are listed here: https://doi.org/10.5281/zenodo.4790455.

Members of the Tree of Life Core Informatics collective are listed here: https://doi.org/10.5281/zenodo.5013541.

Members of the Darwin Tree of Life Consortium are listed here: https://doi.org/10.5281/zenodo.4783558.
