# Supplementary material for: The genome sequence of the surf clam, Spisula solida (Linnaeus, 1758)
Source: Wellcome Open Res. 2023 May 24;8:227. [Version 1] doi: 10.12688/wellcomeopenres.19486.1 (PMC11101916; doi:10.12688/wellcomeopenres.19486.1)
Supplement: Peer review history

# Reviewer report: Reviewer response for version 1

**DOI:** 10.21956/wellcomeopenres.21584.r76961

**Published:** 2025-05-27

**Authors:** David Osca

**Affiliations:** 1 Institute of Environmental Studies and Natural Resources, University of Las Palmas de Gran Canaria Faculty of Marine Sciences (Ringgold ID: 201459), Las Palmas de Gran Canaria, Canary Islands, Spain

Dear Author,

 Firstly, I would like to commend you on the effort put into sequencing the genome of *Spisula subtruncata* (Linnaeus, 1758). It is well-known that there is a significant gap in knowledge regarding molluscs, which obstructs evolutionary studies and genome annotations. Therefore, any new information is highly valuable. Also, the genetic organization in molluscs is remarkably diverse, even within classes and superfamilies at the mitogenomic level, making any additional data very appreciated.

 The quality of the assembly, with a high N50, is noteworthy. However, the annotation is somewhat lacking and could potentially be improved by use the BUSCO database for example eukaryotic organisms.

 Regarding the background, I miss scientific references describing/identifying the species, as well as images that confirm the identification of the specimen. The images in Figure 1 do not have enough quality to be used in a paper; the organism is not correctly oriented and one of the photographs is even overexposed and blurry.

 Concerning the mitochondrial genome, it is mentioned in the methodology and Table 1, but there is no reference to its genetic organization or any characteristics in the text. There is also no explanatory figure at least.

 In the methodology, the references for the kits used, as well as the equipment, are not correctly cited. Lastly, there is an error in the Data Availability section, referring to Table 1 for the raw data and assembly accession identifiers, without mentioning Table 2, which contains the INSDC accession numbers.

 The article should include all these corrections.

Are sufficient details of methods and materials provided to allow replication by others?

Yes

Is the rationale for creating the dataset(s) clearly described?

Yes

Are the datasets clearly presented in a useable and accessible format?

Partly

Are the protocols appropriate and is the work technically sound?

Yes

Reviewer Expertise:

Evolutionary biology, barcoding, phylogenetics, Integrative taxonomy, molecular data, genetics

I confirm that I have read this submission and believe that I have an appropriate level of expertise to confirm that it is of an acceptable scientific standard, however I have significant reservations, as outlined above.

# Reviewer report: Reviewer response for version 1

**DOI:** 10.21956/wellcomeopenres.21584.r71340

**Published:** 2024-05-26

**Authors:** Zhongming Huo

**Affiliations:** 1 Dalian Ocean University, Dalian, Liaoning, China

The chromosome-level genome sequence of the surf clam was reported in this article. The author showed a final assembly with a total length of 932.1 Mb. Did this meet expectations? The author did not provide an understanding of the genome of this species in the background, such as chromosome number, genome size, etc. So, it's unclear whether this assembly is close to the real genome size of the surf clam. Hi-C data showed that the assembly had 19 chromosome-scale scaffolds. Is this consistent with the real chromosome number ? And the quality of annotations is not very good. Complete BUSCOs (C) was not high. Only 13,833 protein-coding genes were annotated. The author needs to do more work to annotate this genome.

Are sufficient details of methods and materials provided to allow replication by others?

Partly

Is the rationale for creating the dataset(s) clearly described?

Partly

Are the datasets clearly presented in a useable and accessible format?

Partly

Are the protocols appropriate and is the work technically sound?

Yes

Reviewer Expertise:

genome, genetic breeding

I confirm that I have read this submission and believe that I have an appropriate level of expertise to state that I do not consider it to be of an acceptable scientific standard, for reasons outlined above.

# Reviewer report: Reviewer response for version 1

**DOI:** 10.21956/wellcomeopenres.21584.r80926

**Published:** 2024-05-17

**Authors:** Taro Maeda

**Affiliations:** 1 Keio University, Keio, Japan

I appreciate the opportunity to review this manuscript. Bivalve genomic information is quite scarce, making this paper particularly valuable for its coverage of non-sessile bivalve genomes. The N50 is impressively high, and it appears that chromosome-level sequences were obtained. Given the difficulty of DNA extraction in this group, these results are certainly noteworthy enough to merit reporting.

 An omission in this manuscript is the lack of mention of the total genome size. The authors note that the sequence data represents 31-fold coverage, but no clear basis for the genome size estimate is provided. Additionally, the total amount of sequence in base pairs is not reported.

 The total number of genes is relatively low for a eukaryote, and the BUSCO scores are surprisingly low given the length of the assembly. This may reflect unique biological characteristics of the organism, possibly biological factors such as parasitism, which may influence the genomic gene number. If the authors could provide descriptions of whether such biological backgrounds exist for this species, it would enhance the readability and understanding of the paper. At this point, I do not know if there are such characteristics in this species that could cause such a genomic feature. If so, the lack of a clearly stated genome size combined with this could give the impression that the sequenced genome may be a partial subset missing significant gene regions.

Are sufficient details of methods and materials provided to allow replication by others?

Yes

Is the rationale for creating the dataset(s) clearly described?

Yes

Are the datasets clearly presented in a useable and accessible format?

Yes

Are the protocols appropriate and is the work technically sound?

Yes

Reviewer Expertise:

molluscan genomics

I confirm that I have read this submission and believe that I have an appropriate level of expertise to confirm that it is of an acceptable scientific standard.

# Reviewer report: Reviewer response for version 1

**DOI:** 10.21956/wellcomeopenres.21584.r80929

**Published:** 2024-05-09

**Authors:** Marco Gerdol

**Affiliations:** 1 Department of Life Sciences, University of Trieste, Trieste, Italy

This manuscript reports the genome assembly of the surf clam *Spisula solidissima*. This is another nice addition by the Darwin Tree of Life project to the growing list of high quality bivalve genomes available. Since the genome has been assembled using well established protocols, I have no technical concerns, and the assembly metrics definitely support the high quality of this resource. The gene annotation is, however, largely incomplete, and I would suggest the authors to try to use an alternative to the Ensembl rapid pipeline, which performs rather poorly for bivalves, when no RNA-seq data is available.

 “Gene annotation of this assembly on Ensembl identified 13,833 protein coding genes.”

 This seems an extremely low number of genes compared with expectations for bivalves and a fast check of the gene models available through the Ensembl rapid annotation web portal indicates that the annotation is largely incomplete. Although making a gene annotation track available to the scientific community is a great addition, I would urge the authors to improve this aspect, considering the high complexity of bivalve genomes and the limited usefulness of such an incomplete annotation track, which could be most definitely largely improved by the use of a standard annotation pipeline, such as BRAKER3.

 Does the size of the assembly match estimated c-value (if available)? Does the number of scaffolded pseudochromosomes match the expected number of metaphasic chromosome pairs (if this data is available for this species)?

 The mollusca_odb10 reference set is notoriously inadequate for estimating the completeness of bivalve genomes, due to its reliance on a very low number of molluscan species (mostly gastropods). I would suggest the authors to report the BUSCO scores obtained against the metazoan dataset as well.

 Genome annotation report: as previously said, the annotation did perform rather poorly. This is not a specific issue of this genome, since pretty much all the other bivalve genomes annotated using this pipeline suffer from similar issues, suggesting that the Ensembl rapid pipeline has some performance issues with bivalve genomes, in particular when protein alignment data only is used (i.e. without RNA-seq data), as it is the case of this genome.

Are sufficient details of methods and materials provided to allow replication by others?

Yes

Is the rationale for creating the dataset(s) clearly described?

Yes

Are the datasets clearly presented in a useable and accessible format?

Yes

Are the protocols appropriate and is the work technically sound?

Partly

Reviewer Expertise:

Non-model species genomics

I confirm that I have read this submission and believe that I have an appropriate level of expertise to confirm that it is of an acceptable scientific standard, however I have significant reservations, as outlined above.

# Reviewer report: Reviewer response for version 1

**DOI:** 10.21956/wellcomeopenres.21584.r71344

**Published:** 2024-01-24

**Authors:** Daniel Garcia-Souto

**Affiliations:** 1 University of Santiago de Compostela, Santiago de Compostela, Galicia, Spain

Dear authors,

 I've had the opportunity to review the manuscript entitled "The genome sequence of the surf clam, *Spisula solida *(Linnaeus, 1758)", authored by Holmes et al. I commend the authors for their significant contribution, particularly in achieving a genome assembly of *Spisula solida* at the chromosome level, accompanied by commendable N50 metrics. Here they present their results on the genome assembly of *Spisula solida*, assembled to chromosome level and achieving magnificent N50 metrics. Moreover, it's always noteworthy highlighting the context of assembling genomes in this taxonomic group (bivalves), largely unrepresented in genomic analysis despite their biological relevance and (usually) posing a great challenge due to their intrinsic DNA quantity/quality issues. As for the manuscript itself, it's very correctly elaborated, both from the methodological and written points of view. I wanted to express some concerns/suggestions in this regard.

 The manuscript itself is impeccably elaborated, demonstrating precision both in its methodology and written presentation. However, I would like to express some considerations and suggestions for further enhancement.

 Primarily, my principal concern revolves around the taxonomic identification of the specimens. While acknowledging the authors' expertise and the inclusion of specimen photographs, these images are insufficient for the rigorous verification of taxonomic identification. I recommend the inclusion of additional photographs depicting the hinge and teeth, as these features serve as crucial diagnostic characters. If the shells are still accessible, kindly consider providing pertinent details in this section.

 The achievement of encapsulating the entire genome within 126 haploid contigs is indeed remarkable. However, I would like to inquiry about the handling of the "purged duplicates." Given that these organisms typically contain extensive heterochromatin blocks comprising highly repeated, low-variability satellite DNAs, it is common practice to exclude them from the definitive genome version. This exclusion may influence final metrics such as ATGC composition, satellite/transposable element content, or even BUSCO completeness scores, as certain repetitive gene families might also be omitted. I kindly suggest the authors to verify whether these contigs were excluded from the final genome version with a brief sentence.

 Finally, and despite the chromosome assembly level of their genome, to my surprise, the BUSCO completeness report for Molluscan is quite low. Could authors pinpoint to why is this happening?

 In conclusion, beyond these considerations, I find the manuscript to be meticulously prepared.

Are sufficient details of methods and materials provided to allow replication by others?

Yes

Is the rationale for creating the dataset(s) clearly described?

Yes

Are the datasets clearly presented in a useable and accessible format?

Yes

Are the protocols appropriate and is the work technically sound?

Yes

Reviewer Expertise:

Genome assembly, repetitive DNA, molecular cytogenetics, phylogenetics, bivalves

I confirm that I have read this submission and believe that I have an appropriate level of expertise to confirm that it is of an acceptable scientific standard.
